# The Effect of Antisolvent Treatment on the Growth of 2D/3D Tin Perovskite Films for Solar Cells

**DOI:** 10.1021/acsenergylett.4c02745

**Published:** 2024-12-17

**Authors:** Ganghong Min, Robert J. E. Westbrook, Meihuizi Jiang, Margherita Taddei, Ang Li, Thomas Webb, Sanjayan Sathasivam, Amanz Azaden, Robert G. Palgrave, David S. Ginger, Thomas J. Macdonald, Saif A. Haque

**Affiliations:** † Department of Chemistry and Centre for Processable Electronics, Molecular Sciences Research Hub, Imperial College London, London W12 0BZ, U.K.; ‡ Department of Chemistry, University of Washington, Seattle, Washington 98195, United States; § Advanced Technology Institute, Department of Electrical and Electronic Engineering, University of Surrey, Guildford, Surrey GU2 7XH, U.K.; ∥ School of Engineering, London South Bank University, London SE1 0AA, U.K.; ⊥ Department of Chemistry, University College London, London WC1H 0AJ, U.K.; # Department of Electronic and Electrical Engineering, University College London, London WC1E 7JE, U.K.

## Abstract

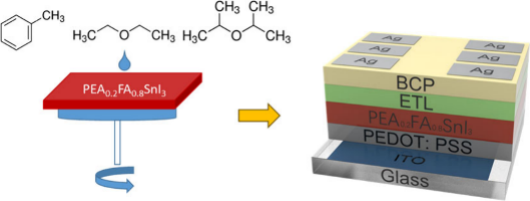

Antisolvent treatment is used in the fabrication of perovskite films to control grain growth during spin coating. We study widely incorporated aromatic hydrocarbons and aprotic ethers, discussing the origin of their performance differences in 2D/3D Sn perovskite (PEA_0.2_FA_0.8_SnI_3_) solar cells. Among the antisolvents that we screen, diisopropyl ether yields the highest power conversion efficiency in solar cells. We use a combination of optical and structural characterization techniques to reveal that this improved performance originates from a higher concentration of 2D phase, distributed evenly throughout the 2D/3D Sn perovskite film, leading to better crystallinity. This redistribution of the 2D phase, as a result of diisopropyl ether antisolvent treatment, has the combined effect of decreasing the Sn^4+^ defect density and background hole density, leading to devices with improved open-circuit voltage, short-circuit current, and power conversion efficiency.

Organic–inorganic hybrid perovskite solar cells (PSCs) have achieved a verified power conversion efficiency (PCE) over 26%.^1^ Significant advances in Pb-based perovskite device stability have also been reported.^2−5^ Furthermore, recent reports have shown dramatic improvements in lead containment,^6−10^ and it has been argued that lead content in perovskite solar cells is dwarfed by the heavy metals in coal consumption.^11,12^ Nevertheless, the use of lead remains an issue of concern for the large-scale manufacture of PSCs. These concerns provide the impetus to continue the search for Pb-free alternatives,^13,14^ with tin (Sn)-only-based PSCs emerging as a well-suited candidate due to the combination of their narrow bandgap, high photoluminescence quantum yield (PLQY) and high carrier mobilities.^15,16^ Despite steady progress in the past few years, the PCE (current best PCE = 15.14%, certified)^17^ of Sn PSCs still remains lower than their Pb counterparts. This discrepancy is due (in part) to the instability of Sn^2+^ which oxidizes to Sn^4+^ already in the precursor solution^18^ and thus cannot be incorporated into the ABX_3_ perovskite structure. Theoretical studies predict that extrinsic Sn^4+^ is stabilized at the surface, behaving as a charge trap, while in the bulk it is reduced back to Sn^2+^, a process which has been suggested to induce background hole doping (eq 1).^19^ Moreover, lattice Sn (Sn_Sn_^0^) is less stable than lattice Pb in APbX_3_ perovskites. This instability leads to the production of more background holes and Sn vacancies (V_Sn_^2–^), the latter of which are linked with the formation of Sn-rich trap clusters (eq 2).^20−23^ Moreover, the presence of such defect states leads to undesirable radiative and nonradiative charge carrier loss processes, which ultimately limit device performance.
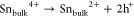
1
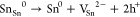
2

To improve the stability of Sn-based halide perovskites, large cations such as PEA have been introduced to form a so-called 2D/3D Sn perovskite.^24−27^ The 2D perovskite phase simultaneously passivates trap states and makes the underlying 3D perovskite phase more resistant to the oxidation that leads to unwanted doping.^28−31^ It is therefore important to develop new fabrication methods that enable the tuning and positioning of the 2D phase within the 2D/3D perovskite film.^32^ Furthermore, interfacing large cations,^33^ regulating self-doping control,^34^ and initiating self-repair via additive engineering have emerged as effective tools to mitigate Sn^2+^ oxidation and trap state formation in the best performing Sn perovskite solar cells.^35−38^

Antisolvent treatments in the Pb perovskite field have played a large part in minimizing V_OC_ losses and therefore improving PCE.^39−41^ Spin coating with antisolvent treatment induces rapid and dense nucleation of the perovskite, leading to uniform and pinhole-free films.^41^ To date, various antisolvents with different boiling points and different polarities have been used for optimizing perovskite fabrication.^42−46^ Studies have shown that the dissolution capacity of an antisolvent for precursor components, along with its miscibility with the perovskite host precursor, are pivotal in the formation of perovskite films.^40^ Importantly, device performance exhibits a strong dependence on these factors. Lead-based perovskites consistently yield superior device performance and reproducibility when antisolvent treatment is employed.^40,47,48^ However, relatively few reports have focused on the role of antisolvent treatment on the crystal growth and minimization of defects in Sn perovskites.^49−52^ Such knowledge is expected to play a key role in the future design and optimization of lead-free Sn-perovskite solar cells.^53^

In this paper, we demonstrate the effect of different antisolvents—toluene, diethyl ether (DE), and diisopropylether (DIE)—on the performance of 2D/3D Sn PSCs. Notably, we omit chlorobenzene because it has significantly poorer performance as an antisolvent when compared to toluene or the ethers, as established in our previously optimized 2D/3D Sn-PSCs.^20,35^ We show that the choice of antisolvent determines the distribution of 2D phase within the 2D/3D Sn perovskite film. We see that DIE-treated films form 2D phases at the top and bottom of the film, resulting in more overall 3D–2D interfaces. We see that the more uniform distribution of 2D regions improves the films crystallinity and stability as measured by a combination of X-ray diffraction and absorption spectroscopy. Sn PSCs made with DIE treatment showed both the highest efficiency and improved stability in ambient air. The lower polarity of DIE makes it immiscible with DMSO, thus fostering the formation of a DMSO-containing intermediate that slows the crystal growth. This new understanding of the link between antisolvent treatment, phase distribution, and device performance will aid the further development of Sn PSCs.

All devices were fabricated as shown in Figure 1a, with the device structure ITO/PEDOT:PSS/Sn perovskite/ETL/BCP/Ag (acronyms defined in the Methods). We tested both PCBM and ICBA as electron transport layers with different energy band levels (Figure 1b).^54^ The Sn perovskite active layer was formed after antisolvent dripping with toluene, DE and DIE. When PCBM is employed as the ETL, the device performance of our champion Sn PSCs treated with each of the different antisolvents is outlined in Table 1 and Figure 1c. DIE treatment yields the best device power conversion efficiency (PCE; 10.14%), followed by DE (7.92%) and toluene (7.45%). We include maximum power point (MPP) tracking in Figure S1, which shows the stable output of the Sn perovskite solar cells treated with different antisolvents. Figure S2 indicates that the Sn perovskite solar cell treated by all antisolvents has a slight hysteresis. We observe the same trend in device performance after the different antisolvent treatments across 12 devices via statistical analysis (Figure 1d, Figure S3). The relatively high PCE of DIE-treated Sn perovskite solar cells is derived from having the highest short-circuit current (*J*_SC_), open-circuit voltage (*V*_OC_) and fill factor (FF) of all the antisolvent treatments. The integrated current density of 19.9 mA/cm^2^ from external quantum efficiency (EQE) measurements (Figure S4) matches the average *J*_SC_ from the *J*–*V* curves, excluding any spectral mismatch as highlighted in previous work.^55,56^ Most notably, the *V*_OC_ after DIE treatment exceeded 0.6 V in all cases and averaged around 0.1 V higher than with the other antisolvents. While the *J*_SC_ and FF are largely maintained in DE-treated devices, the *V*_OC_ is significantly lower, yielding a poorer power conversion efficiency of 7.9%. In toluene-treated devices, the *J*_SC_, FF and *V*_OC_ are further reduced, resulting in a champion PCE of 7.45%. We further optimized the device performance by replacing PCBM with ICBA for better band alignment with Sn perovskite.^57^ With the employment of ICBA as the electron transporting layer, DIE-treated Sn perovskite achieved a PCE of 13.52%, higher than DE-treated (10.47%) and toluene-treated (9.04%) Sn perovskite (Table 1, Figure 1e and Figure 1f). Similar to PCBM-based devices, DIE-treated Sn perovskite consistently reveals higher *V*_OC_, *J*_SC_ and FF among other antisolvents (Figure S5). These observations further indicate the positive influence of the DIE antisolvent treatment on device performance.

**Figure 1:**
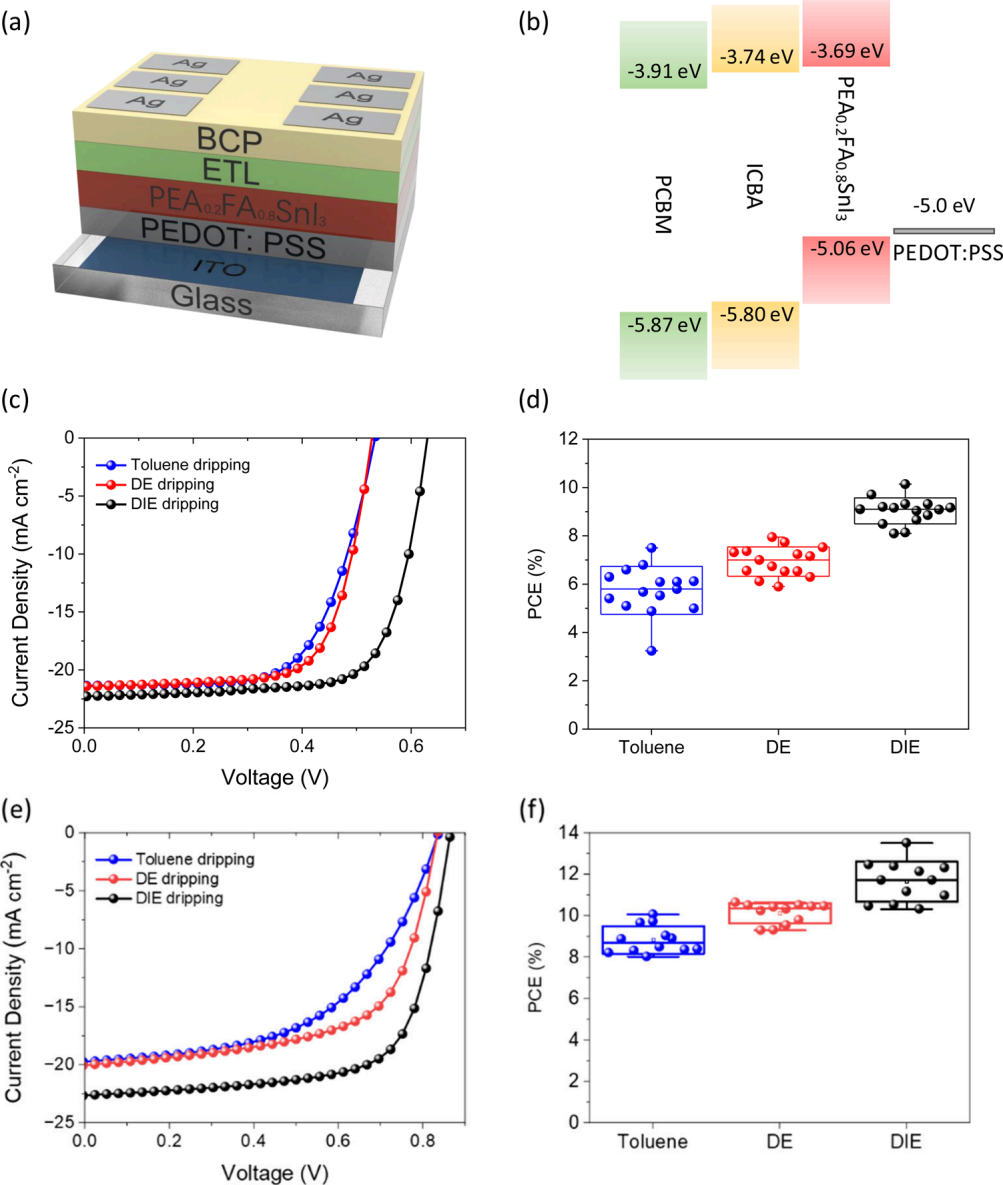
(a) Schematic structure of the p-i-n structured PEA_0.2_FA_0.8_SnI_3_ solar cells. (b) Energy level diagram of PCBM, ICBA, PEA_0.2_FA_0.8_SnI_3_ and PEDOT:PSS, indicating the highest-occupied molecular orbital (HOMO) and lowest-unoccupied molecular orbital (LUMO). Values of PCBM and ICBA were extracted from literature reports that used cyclic voltammetry.^52,55^ The values of PEA_0.2_FA_0.8_SnI_3_ were determined by UPS measurement and UV–vis spectroscopy (Figure S6). The values given in the figure are in eV. (c) The *J*–*V* curves of champion PCBM-based devices made with the different antisolvents under the AM1.5 solar spectrum at an intensity of 1 sun with a scan rate of 50 mV/s. All *J*–*V* curves were collected as forward scans. (d) Statistical data on power conversion efficiency of PCBM-based devices for each antisolvent treatment. (e) The *J*–*V* curves of champion ICBA-based devices made with the different antisolvents under the AM1.5 solar spectrum at an intensity of 1 sun with a scan rate of 50 mV/s. All *J*–*V* curves were collected as forward scans. (f) Statistical data on power conversion efficiency of ICBA-based devices for each antisolvent treatment.

**Table 1:** Photovoltaic Parameters, Extracted from Forward *J*–*V* Scans, of Sn Perovskite Solar Cells Treated with Three Antisolvents^a^

Antisolvent/ETL	*V*_OC_/V	*J*_SC_/mA cm^–2^	FF/%	PCE/%
DIE/PCBM	0.614 ± 0.010	20.97 ± 0.62	68.91 ± 1.67	9.04 ± 0.54
	**0.630**	**22.28**	**72.09**	**10.14**
DE/PCBM	0.532 ± 0.017	20.05 ± 1.67	65.03 ± 4.53	6.93 ± 0.60
	**0.528**	**21.41**	**70.06**	**7.92**
Toluene/PCBM	0.521 ± 0.015	16.94 ± 2.31	63.03 ± 3.61	5.74 ± 0.99
	**0.534**	**21.37**	**65.14**	**7.45**
DIE/ICBA	0.847 ± 0.018	20.94 ± 1.63	65.93 ± 1.99	11.65 ± 0.94
	**0.865**	**22.65**	**69.20**	**13.52**
DE/ICBA	0.807 ± 0.018	20.25 ± 1.27	62.41 ± 1.61	10.13 ± 0.47
	**0.835**	**20.04**	**62.73**	**10.47**
Toluene/ICBA	0.774 ± 0.019	19.73 ± 0.97	57.92 ± 2.21	8.84 ± 0.64
	**0.837**	**19.73**	**54.74**	**9.04**

a We include the mean and standard deviation across 15 devices based on PCBM and ICBA in 1 batch respectively along with the champion device parameter in bold.

We employed a series of characterization techniques to assess the optical and structural properties of the Sn perovskite films treated with the different antisolvents. We first used X-ray Diffraction (XRD) to directly probe the differences in grain growth after treatment with toluene, DE and DIE. The XRD pattern in Figure 2a matches well with previous reports of Sn perovskites. We see a feature at 2θ = 14° and 28° corresponding to the (001) and (002) orientations of 3D Sn perovskite consistent with an orthorhombic crystal structure (*Amm*2 space group) as expected.^20,58^ The intensity of the peaks of Sn perovskite films treated by DIE is approximately double that of films processed with DE and toluene, despite similar film thickness (as investigated by profilometry; see Table S1). Table S2 lists the crystallite size of Sn perovskite determined from the FWHM of (001) peak, which is largest for the DIE-treated Sn perovskite, suggesting it has the larger crystallite sizes. Furthermore, we also applied grazing incidence XRD (GIXRD) to obtain crystallographic information specific to the film surface. The GIXRD patterns in Figure 2b consist of the familiar (001) peak at 2θ 14° and a new peak at 4.0°, which corresponds to (PEA)_2_(FA)Sn_2_I_7_.^59^ It is clear from Figure 2b that the concentration of 2D phase at the surface increases in the order [toluene < DE < DIE]. Taken together, the XRD and GIXRD results show that films that exhibit a greater amount of 2D phase at the surface also have a greater concentration of crystalline material in the 3D phase. Figure S7 shows the morphologies (from scanning electron microscopy) of each Sn perovskite treated with the different antisolvents, indicating that DIE-treated Sn perovskite has a smooth surface and the best coverage.

**Figure 2:**
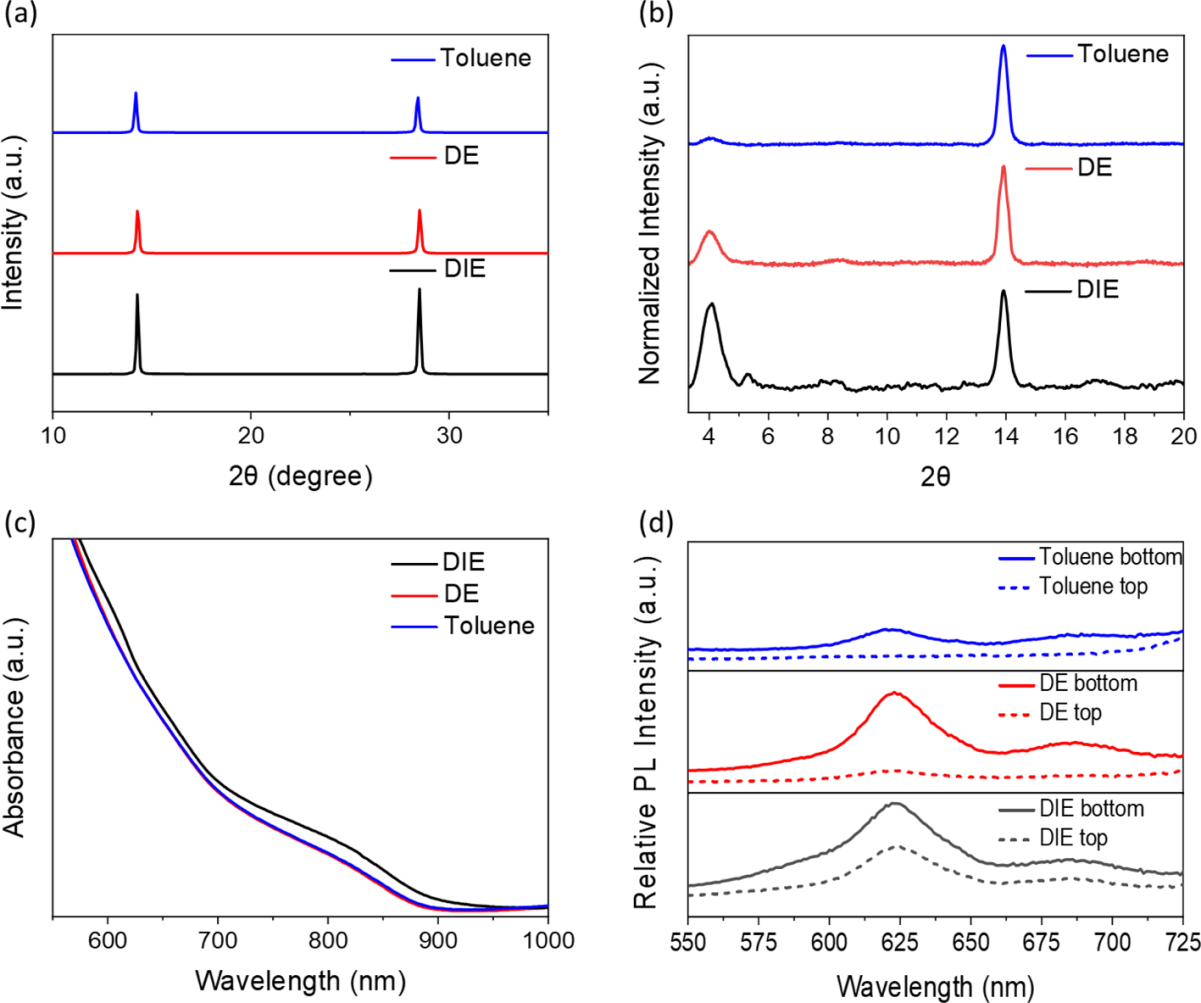
(a) XRD patterns of PEA_0.2_FA_0.8_SnI_3_ films treated by three different antisolvents. (b) The normalized intensity of GIXRD patterns of perovskite films prepared with three different antisolvents at an incident angle of 2.5°. (c) UV–vis spectra of PEA_0.2_FA_0.8_SnI_3_ films treated by three different antisolvents. (d) Comparison of photoluminescence emission spectra of perovskite films treated by three different antisolvents.

The UV–vis absorption data in Figure 2c shows that DIE-treated Sn perovskite films have a more pronounced band edge than the DE- or toluene-treated Sn perovskite films. We rule out increased film thickness as responsible for this, given the similar thickness of the different films (Table S1). Additionally, the DIE-treated Sn perovskite has higher absorbance around 610 nm, consistent with the formation of a greater fraction of 2D phase in this film compared with those after DE or toluene treatment.^60^

We next probed the concentration and distribution of 2D phases within the 2D/3D Sn perovskite with photoluminescence (PL) spectroscopy. We used an excitation wavelength of 450 nm, which has a penetration depth—defined as the distance upon which the incident light intensity is reduced to 1/e of its initial intensity—of 50 nm, allowing us to bias the excitation profile toward the bottom (substrate/perovskite interface) or top (perovskite/encapsulating glass interface) surfaces of the Sn perovskite film (thickness = 200 ± 10 nm, Table S1). This allowed us to obtain local emission spectra representative of the 2D concentration in those locations. The PL spectrum of Sn perovskite films processed with DIE in Figure 2d exhibits a strong emission at 620 nm which represents 2D perovskite (PEA_2_SnI_4_).^24,61^ Notably, the emission intensity from this peak is similar regardless of the excitation surface, suggesting that the 2D phase is evenly distributed throughout the vertical axis of the film. After DE treatment, the PL is still intense at the bottom surface, but the intensity at the top surface is much lower. This suggests that most of the 2D phase in DE-treated Sn perovskite is concentrated at the substrate/perovskite interface. In films processed with toluene, only weak 2D phase emission can be observed after excitation of the bottom surface with no emission at all observed after excitation of the top surface. The lower PL intensity at 620 nm, concentrated at the bottom surface, suggests that (i) there is less 2D phase formed overall and (ii) the 2D phase is concentrated to the substrate/perovskite interface of the film.

We further probed the distribution of the 2D phase within the 2D/3D Sn perovskite film with hyperspectral PL microscopy (Figure 3). In this experiment, we captured 40 × 50 μm images of the Sn perovskite emission at 620 nm—representative of the 2D perovskite, PEA_2_SnI_4_—after excitation from the top and bottom surfaces of the film. As before, here “top” is defined as the perovskite/encapsulating glass interface and “bottom” is defined as the “perovskite/substrate interface”. In Figure 3a–c we show the 2D phase emission from the top of the films processed with toluene, DE and DIE. We see that the film processed with toluene shows sparsely distributed clusters of 2D phase. Processing with DE improves the 2D phase coverage and intensity by over 6 times, and the DIE by 75 times. In Figure 3d–f we show the distribution of 2D phase after excitation from the bottom of the films. The 2D phase in the bottom is brighter than the top, as expected from the previous PL spectroscopy data. From hyperspectral microscopy we see the formation of larger 2D regions. Figure 3 provides new insight into the lateral distribution of 2D phase in the different films, which becomes more dense in the order toluene < DE < DIE.

**Figure 3:**
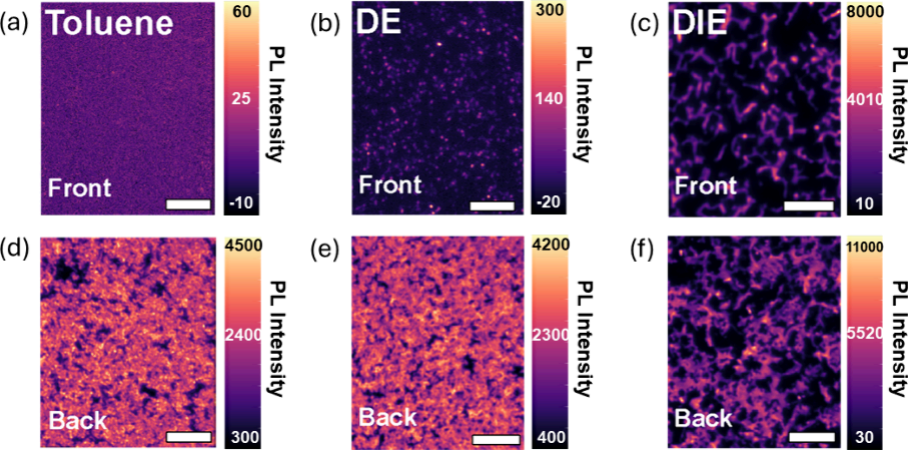
(a–c) Hyperspectral microscopy images, recorded at 620 nm from the “front” side (perovskite/encapsulating glass interface), for PEA_0.2_FA_0.8_SnI_3_ treated with (a) toluene, (b) diethyl ether (DE), and (c) diisopropyl ether (DIE). (d–f) Hyperspectral microscopy images, recorded at 620 nm from the “back” side (perovskite/substrate interface), for PEA_0.2_FA_0.8_SnI_3_ treated with (d) toluene, (e) diethyl ether (DE), and (f) diisopropyl ether (DIE). The scale bars in all cases represent 20 μm.

Ultimately, these data connect the macroscopic device parameters with the microscopic arrangement of the 3D and 2D components of the perovskite film. We propose that the poorer *V*_OC_ perovskite cells processed with DE and toluene are due to the lower concentration of 2D phase within the 2D/3D perovskite film in these cases. The lower concentration of the 2D phase will have less passivating power over the 3D component, rendering it prone to trap state formation and self-doping. On the other hand, the even distribution of the 2D component after processing with DIE will render those films with extensive passivation throughout the film, rather than just at one interface. Additionally, the improved distribution of 2D phase within the 2D/3D perovskite film in DIE-treated films may better template the growth and orientation of the 3D phase.^62^

Next, we investigated the chemistry of the precursor solution in an attempt to identify the origin(s) of the different film composition following antisolvent treatment with toluene, DE and DIE. Figure S8 shows images of PEA_0.2_FA_0.8_SnI_3_ precursor solutions (DMF:DMSO = 4:1) both initially and after 6 h of mixing with each antisolvent. Herein we find that DIE is not miscible with the precursor solution system at the selected concentrations. This lack of miscibility is evidenced by the precipitation of the precursors upon mixing and the phase separation between the solvents and antisolvent after 6 h. As has been found for Pb perovskites, a high degree of miscibility between antisolvent and precursor solvents may lead to the removal of DMSO during the film formation process, which in turn will preclude the formation of the intermediate DMSO complex needed for slow growth of high-quality perovskite crystals. We note that the immiscibility between DIE and the precursor solution is likely to lead to reduced loss of PEA during the antisolvent dripping process thus resulting in more 2D phase in the final 2D/3D Sn perovskite film.^39^ We include details of the relative polarity and boiling points of toluene, DE and DIE in Table S3.

We carried out proton NMR to further demonstrate the relationship between the miscibility of the precursor solvent (DMF/DMSO, 4:1) and the composition of the final perovskite films (Figure S9). We conducted the NMR study on films dissolved in deuterated DMSO (DMSO-*d*_6_; see experimental section for further details). From the NMR spectra, we note the existence of FAI (peak positioned at −9.05 ppm and −8.70 ppm) and PEAI (−7.25 ppm and −7.35 ppm) in the dissolved films. By comparing the area ratio of the PEAI and FAI peaks (see Table S4), we found that the Sn perovskite processed with DIE has the highest stoichiometric ratio of PEAI in the film. This finding indicates that less PEAI was washed away by DIE during antisolvent treatment, as compared to DE and toluene, which should induce the formation of more 2D perovskite in the DIE-processed films. We posit that the larger amount of PEAI in the film after DIE treatment is due to the poor miscibility between DIE and DMF/DMSO (4:1).

Next, we used X-ray photoelectron spectroscopy (XPS) to investigate how the different antisolvents affected Sn^4+^ content on the surface of the tin perovskite films. To rule out any side reactions influenced by the presence of moisture and oxygen, we prepared all samples for XPS in a nitrogen glovebox and transferred to the XPS chamber without exposure to air using an inert transfer system. We loaded all of the samples together in the same chamber, meaning they were all subject to the same conditions. In addition, we used the same number of XPS measurement scans for each film to ensure all X-ray exposure times were equal. We observed Sn^2+^ at binding energies 485.8 ± 0.1 eV and 494.8 ± 0.1 eV for Sn 3d_3/2_ and Sn 3d5/2 (respectively), which are in good agreement with literature.^63^ Each Sn 3d peak contained a notable shoulder at slightly higher binding energies (487.1 ± 0.5 eV for Sn 3d_3/2_ and 495.5 ± 0.5 eV for Sn 3d_5/2_) and, upon fitting, we identified this as Sn^4+^. The XPS spectra for DE-, DIE- and toluene-treated films are shown in Figure 4a. In addition, we have calculated the Sn^4+^/Sn^2+^ atomic ratios for each antisolvent treatment where DIE and DE treatment leads to the lowest surface Sn^4+^ content of 8.7 (±0.7)% and 9.7 (±0.8), respectively, whereas toluene treatments lead to the highest Sn^4+^ content of 11.8 (±0.7)%. Our previous report identifies Sn^4+^ states to be detrimental to device PCE, and this is further verified here with our DIE-treated devices showing the highest PCE and lowest Sn^4+^ content.^20,64−67^

**Figure 4:**
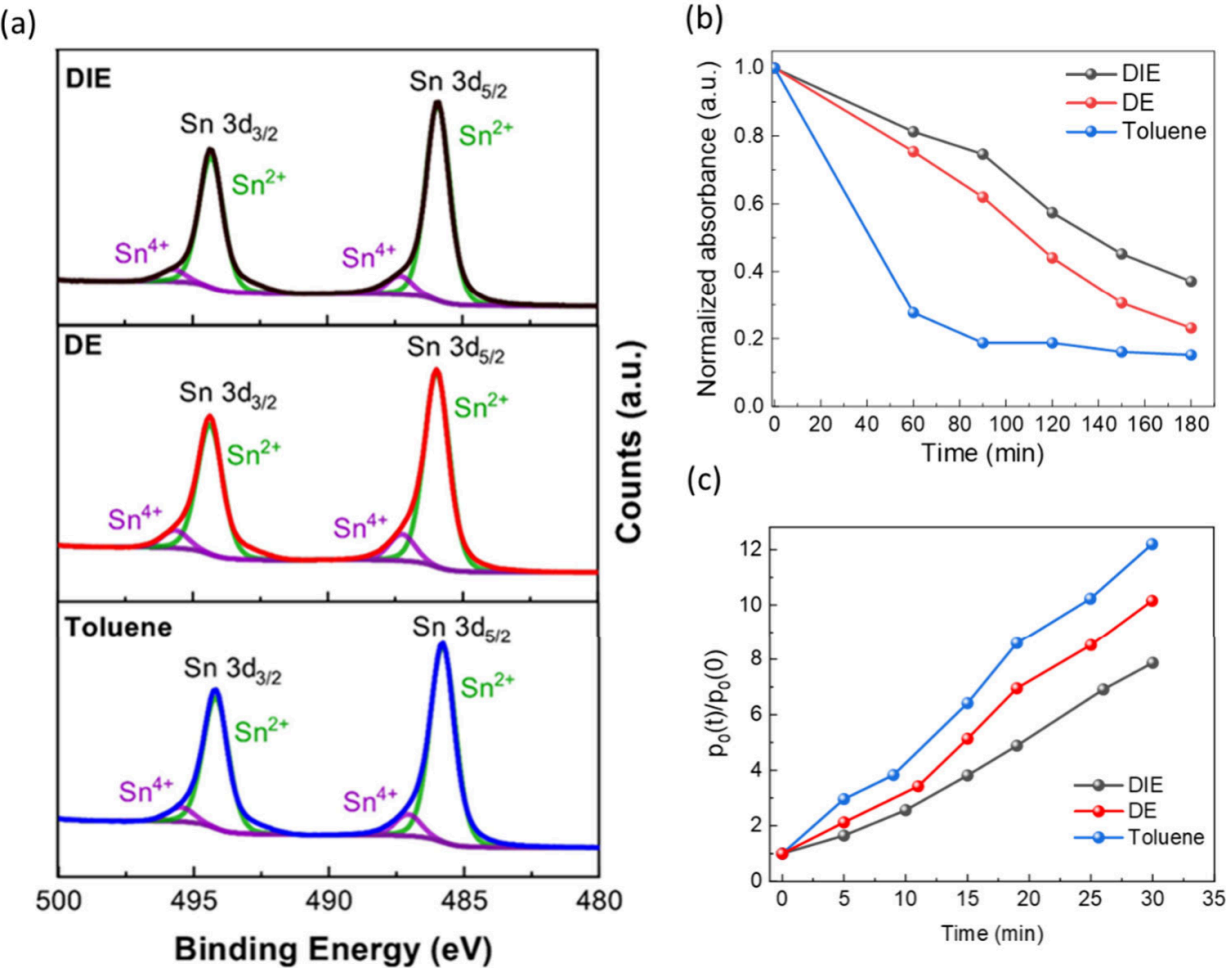
(a) X-ray photoelectron spectra (XPS) for Sn 3d surface scans on the perovskite films for DE (top), DIE (middle) and toluene (bottom) antisolvent treatments. The Sn^4+^ peaks are represented in purple and are seen as shoulders at slightly higher binding energies with each of the main Sn^2+^ 3d peaks (green). (b) Absorbance at 860 nm for perovskite films after the different antisolvent treatments as a function of exposure to ambient atmosphere in the dark. (c) Relative background hole density of 2D/3D tin perovskite films as a function of exposure to ambient atmosphere in the dark, as determined by a time-correlated single photon counting experiment.

Given the observed variations in 2D content and distribution within the films treated with different antisolvents, and the less studied (compared to Pb^2,68,69^), positive impact of 2D functionalization on environmental stability of Sn perovskites, we next turned our focus to the stability of the tin perovskite photoactive layers. Atmospheric oxidation of Sn perovskites induces strong *p*-doping, which ultimately leads to degradation of device performance via unwanted monomolecular radiative recombination.^70^ We evaluated the stability of 2D/3D Sn perovskite films made with the three antisolvents by comparing the absorbance change over time in ambient conditions, shown in Figure 4b. After 60 min, the DIE-treated film maintained 81.2% absorbance, which is slightly higher than the DE-treated film (75.4%), but much higher than toluene (27.7%)-treated films. The original UV–visible absorbance spectra are shown in Figure S10.

In order to connect the differences in stability to changes in doping levels associated with Sn oxidation, we used time-correlated single photon counting (TCSPC) to estimate the relative background hole density, *p*_0_ (see Supplementary Note 1, Figure S11).^71^Figure 4c shows the relative *p*_0_—defined as *p*_0_(*t*)/*p*_0_(0)—as a function of time under exposure to ambient atmosphere in the dark. We find that the increase of *p*_0_ as a function of time is different depending on the antisolvent. Importantly, we observe that the increase in *p*_0_ is lowest in DIE-treated films compared to DE- and toluene-treated films. This measurement shows that DIE treatment offers enhanced resistance to spontaneous p-type doping compared to DE and toluene treatment. We propose that this is due to the better distribution of 2D phase in the 2D/3D film after DIE treatment.

To summarize, we have studied the effects of three different antisolvents (toluene, DE and DIE) on the growth of 2D/3D Sn perovskite (PEA_0.2_FA_0.8_SnI_3_). Sn perovskite processed with DIE shows the best device performance among these three antisolvents, resulting in PCEs of over 10 and 13% for PCBM- and ICBA-based devices, respectively. Compared to the other two antisolvents, DIE treatment leads to the formation of a higher concentration of 2D phase, resulting in a more orientated 3D phase and higher stability to Sn^2+^ oxidation in ambient atmosphere. Comparison of the miscibility of the antisolvents with the precursor solvents revealed that DIE is immiscible with DMSO. This is beneficial for the formation of the intermediate phase, which in turn promotes the crystallization of Sn perovskite. The results of this study indicate the importance of the miscibility between the antisolvent and the perovskite precursor. As such, it is reasonable to suppose that the successful implementation of this antisolvent engineering strategy will inevitably require consideration of both the precursor chemistry and perovskite composition. Finally, the present findings show that antisolvent engineering provides a powerful route to controlling the 2D phase in 2D/3D tin perovskite films. This work should provide new opportunities to boost the performance of lead-free tin perovskite optoelectronics.

## Supporting Information Available

Experimental methods including materials, devices fabrication and all characterization details; thickness measurements, MPP tracking, hysteresis test, statistical data for PV parameters, EQE, device parameters, UPS of perovskite film, FWHM, SEM images, miscibility test, physical characteristics of each antisolvent, NMR spectra of perovskite films, peak area ratios, UV–vis spectra, additional notes of hole density analysis, PS decays, and references (PDF)
