# Supplementary material for: The Effect of Antisolvent Treatment on the Growth of 2D/3D Tin Perovskite Films for Solar Cells
Source: ACS Energy Lett. 2024 Dec 17;10(1):254–62. doi: 10.1021/acsenergylett.4c02745 (PMC11731394; doi:10.1021/acsenergylett.4c02745)
Supplement: Supplementary file 1 — nz4c02745_si_001.pdf [file nz4c02745_si_001.pdf]

Supporting Information:

The Effect of Antisolvent treatment on the Growth of 2D/3D Tin Perovskite films for Solar Cells

Ganghong Min[1], Robert J. E. Westbrook[1, 2], Meihuizi Jiang[1], Margherita Taddei[2], Ang Li[1], Thomas Webb[1,3], Sanjayan Sathasivam[4,5], Amanz Azaden[1], Robert G. Palgrave[5], David S. Ginger[2], Thomas J. Macdonald[1,6*] and Saif A. Haque[1*]

[1]*Department of Chemistry and Centre for Processable Electronics, Molecular Sciences Research Hub, Imperial College London, London W12 0BZ, UK*

[2]*Department of Chemistry, University of Washington, Seattle, WA, 98195 USA*

[3]*Advanced Technology Institute, Department of Electrical and Electronic Engineering, University of Surrey, Guildford, Surrey GU2 7XH, UK*

[4]*School of Engineering, London South Bank University, London, SE1 0AA, UK*

[5]*Department of Chemistry, University College London, London, WC1H 0AJ, UK*

[6]*Department of Electronic and Electrical Engineering, University College London, London WC1E 7JE, UK*

Corresponding authors:

T.J.M (tom.macdonald@ucl.ac.uk) and S.A.H (s.a.haque@imperial.ac.uk)

Methods

Materials

Formamidinium iodide (FAI) and phenethylammonium iodide (PEAI) were purchased from Greatcell Solar. Tin (II) iodide (SnI_2, 99.99%) and tin (II) fluoride (SnF_2, 99%) were purchased from Sigma Aldrich. Indium tin oxide (ITO) was purchased from Psiotec. Poly(3,4-ethylenedioxythiophene) /poly(styrenesulfonate) (PEDOT: PSS) was purchased from Heraeus Clevios. [6,6]-phenyl-C61-butyric acid methyl (PCBM; 99.5%) was purchased from Lumnetic. Indene-C60 Bisadduct (ICBA, 99.5%) was purchased from 1- Materials. Bathocuproine (BCP) was purchased from Sigma Aldrich. N, N-Dimethylformamide (DMF) and dimethyl sulfoxide (DMSO) were purchased from Acros Organics. Deuterated DMSO (DMSO-d_6) was purchased from VWR. All antisolvents were purchased from Sigma Aldrich.

Perovskite film deposition

a 4:1 v/v mixture of DMF and DMSO were filtered with 0.2 µm polytetrafluoroethylene (PTFE) filters. Next, PEAI, FAI, SnI_2 and SnF_2 powders are weighed in a 0.2:0.8:1:0.1 molar ratio, respectively, to obtain 500 µL of a 0.8M $PEA_{0.2}FA_{0.8}SnI_3$ perovskite precursor solution by heating and stirring at 70°C for 1h. The solutions were deposited on the chosen substrate at 4000 rpm for 20 seconds. At 10th second, 0.5 ml antisolvents (DIE, DE, and toluene) was instantly dripped onto the spinning substrate, followed by 70°C annealing for 20 minutes.

Device Fabrication

ITO substrates (purchased from Psiotec, 1.2 cm x 1.2 cm, 15 Ω/cm^2) were rinsed with acetone and then sonicated in soap, distilled water, acetone and isopropanol sequentially. Then the clean ITO was treated by oxygen plasma for 10 min. The device structure in this work is ITO/PEDOT: PSS/Sn perovskite/PCBM/BCP/Ag. The 2D/3D Sn perovskite $PEA_{0.2}FA_{0.8}SnI_3$ was sandwiched between) PEDOT: PSS as the hole transport layer and PCBM as the ETL. Bathocuproine (BCP) was added between the PCBM and the silver electrode as a buffer layer. The PEDOT: PSS was spin coated on ITO at 6000rpm with 2000 rpm/s acceleration for 30s, and annealed at 140 for 20 min. Then the HTL with ITO were transfer to N_2 filled glovebox immediately. Then the perovskite was deposited on the HTL. PCBM solution (20 mg/ml in chlorobenzene) was spin-coated on perovskite films at 2000 rpm for 30s. The BCP (0.5mg/ml in isopropanol) was spin coated on PCBM at 5500rpm for 30s. For ICBA ETL, the ICBA solution (17 mg/ml in chlorobenzene) was spin-coated on

perovskite films at 2500 rpm for 30s. 100 nm Ag electrode was then thermal evaporated at 10^{-6} mbar on the top of devices. The active area of the pixel was limited to 0.045 cm^2 by using a mask.

Film Characterization

UV-Vis spectroscopy measurements were performed with a Shimadzu UV-2600 integrating-sphere spectrophotometer. X-Ray Diffraction was performed with a PANalytical X'Pert Pro MRD diffractometer by using Cu Kα incident radiation (Ni-filtered) at 40 kV and 40 mA. Scanning electron microscopic (SEM) images were collected on a Zeiss Auriga Field Emission Scanning Electron Microscope operated at 3 kV using an InLens model. Samples for SEM were sputtered with 15 nm Cr before SEM measurement. ^{1}H-NMR measurements were conducted with a 400 MHz Bruker setup and the data was analysed by Topspin software.

XPS

X-ray photoelectron spectroscopy (XPS) was carried out on a Thermo Fisher Scientific K-Alpha instrument, using Al Kα radiation (1486.6 eV). All samples were loaded into a Thermo Fisher Vacuum Sample Module in the glovebox to avoid exposure to air during transfer into the spectrometer chamber. Survey spectra were taken at a pass energy of 200 eV. High resolution scans were carried out for Sn 3d and C 1s with a pass energy of 50 eV. Charge compensation was carried out during measurement using argon ion and electron flood guns. All data was analysed using CasaXPS software. The binding energies were adjusted for charging to adventitious carbon at 284.5 eV.

UPS

UPS was carried out in a Thermo Nexsa spectrometer using a He(I) (21.2eV) photon energy. Spectra were recorded with a pass energy of 2 eV. No charge compensation was used. Samples were biased to -9 V relative to the analyser to allow measurement of the low kinetic energy cutoff. Pt foil was measured in contact with the samples as a measure of Fermi level. The spectral width of the UPS is the energy difference between the low and high kinetic energy cutoffs. The workfunction was calculated as the difference between the photon energy

and the spectral width. The electrical contact of the samples with the Pt foil was verified by ramping the bias from 0 to -9V and observing a 1 eV/V shift of all spectra.

Photoluminescence Spectroscopy and TCSPC

Photoluminescence spectroscopies were collected by Fluorolog 1039 fluorescence spectrometer from Horiba. The excitation light was employed a Xenon lamp and the excitation wavelength is 450 nm. All spectrum data were collected by scanning from 550 nm to 725 nm. TCSPC was measured under a Delta Flex system (Horiba Scientific). Before the PL lifetime measurement, the steady state PL spectrum was conducted by a single counting detector (PPD-900, Horiba scientific). Pulsed laser diodes with wavelengths 467 nm were used as the excitation light source.

Hyperspectral Microscopy

We carried out hyperspectral microscopy with a Photon etc. IMA upright microscope fitted with a 60X objective (Nikon Plan RT, NA 0.7, CC 0-1.2). Excitation was achieved episcopically (from above) with an ultrahigh pressure 130 W mercury halide lamp (Nikon) passing through a 500 nm short-pass filter and emission was collected through a 500 dichroic filter and 550 nm long-pass filter. The power density of the excitation was measured to be 1 W cm^{-2} at the sample position. Measurements were carried out in an inert (N_2) environment and typically took 7 minutes to complete. The Hyperspectral Microscope uses a tuneable Bragg filter to detect emission from the sample at specific wavelengths.

^{1}H-NMR sample preparation

The $PEA_{0.2}FA_{0.8}SnI_3$ films treated by different antisolvents without further annealing were dissolved in 0.75ml DMSO-d_6 and then the solutions were transferred into NMR tubes to sample measurements. ^{1}H-NMR measurements were taken with a 400 MHz Bruker setup and all data was analysed by TopSpin software.

Device Characterization

J-V of solar cell devices were measured by simulated AM1.5 solar light (Oriel Instruments) and a Keithley 2400 source metre at a scan rate of 50 mV/s in both forwarding and reversing bias. Light intensity calibration was carried out with a silicon photodiode. Devices were measured under inert conditions during the measurements inside a homemade measuring chamber filling with the glovebox atmosphere.

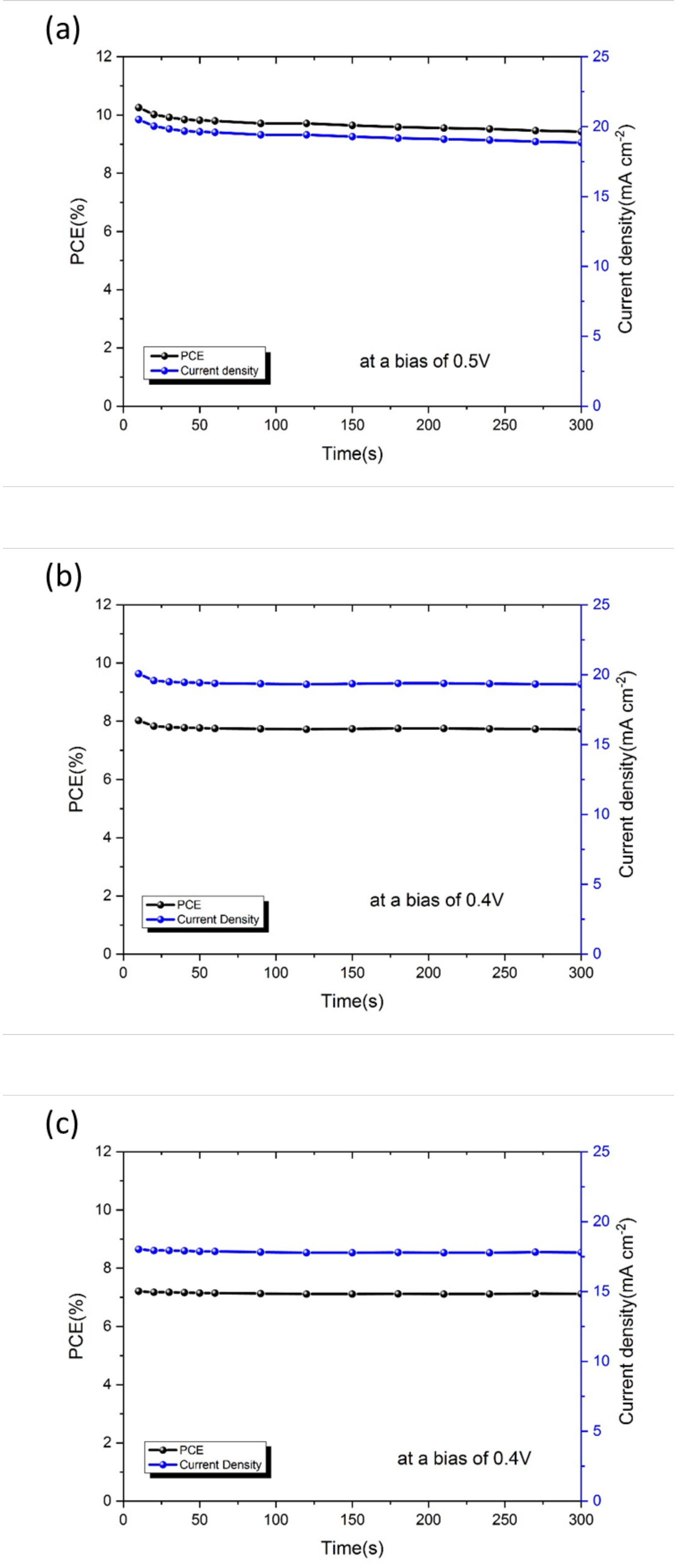

Figure S1. The PCE and the current densities operating at maximum power point (MPP) voltage under illumination (100 mW/cm^{-2}). (a) DIE. (b) DE. (c) Toluene.

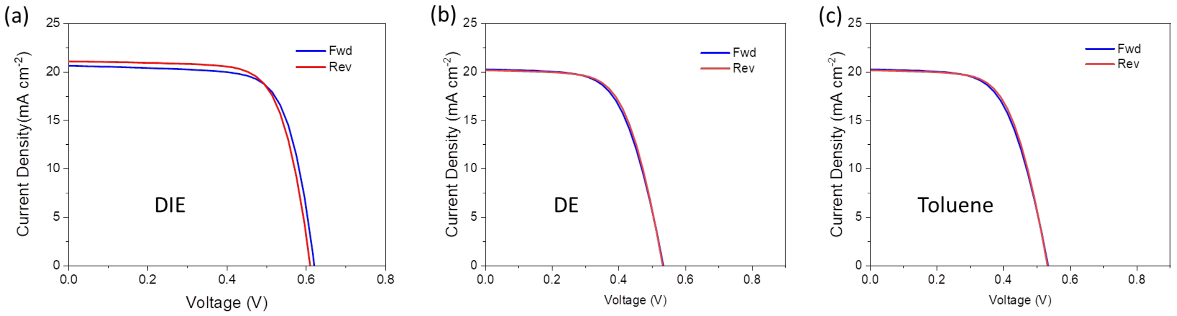

Figure S2. Hysteresis test of Sn perovskite solar cells (PCBM as ETL) with each antisolvents

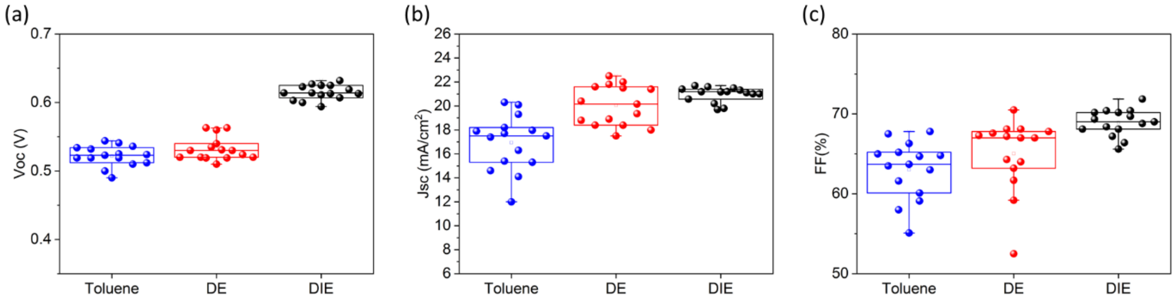

Figure S3. Statistical data on (a) V_{oc}, (b) J_{sc} and (c) FF based on PCBM ETL

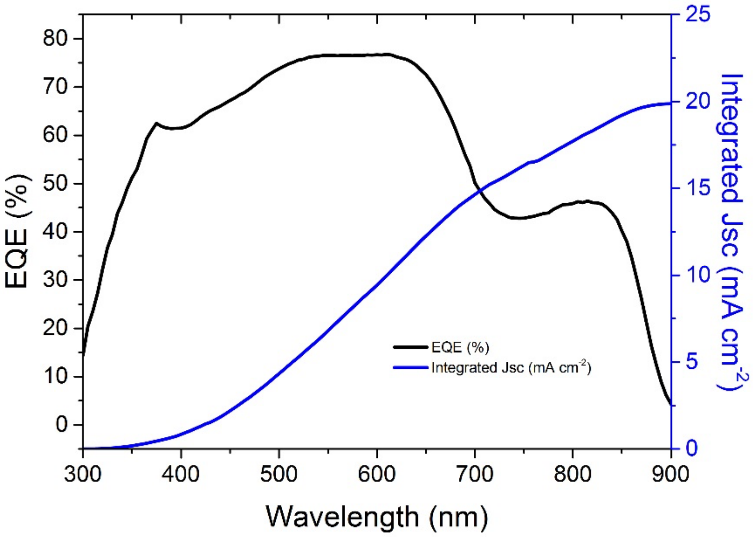

Figure S4. The EQE spectra of the DIE treated Sn perovskite solar cell based on PCBM ETL. The mismatch of Jsc might attributed to that the measuring range is not fully covered.

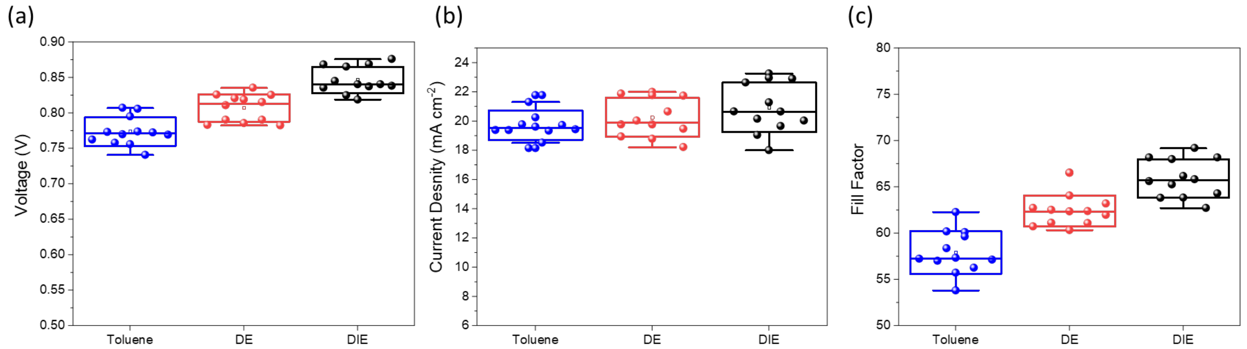

Figure S5. Statistical device parameters on (a) V_{oc}, (b) J_{sc} and (c) FF based on ICBA ETL.

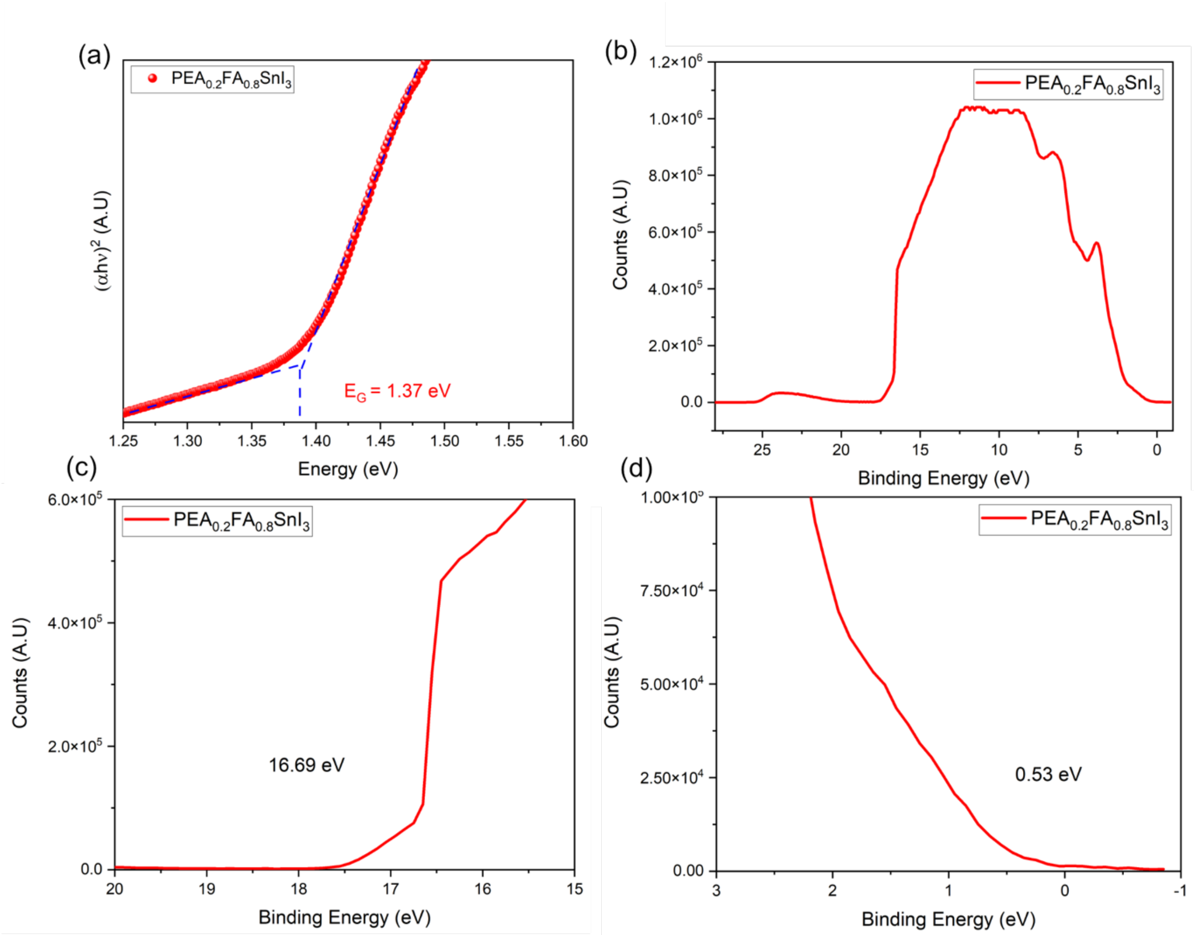

Figure S6. The (a) Tauc Plot of Sn perovskite determining the bandgap is 1.37 eV. (b) The whole UPS spectrum,(c) energy cutoff region and (d) energy onset region of UPS spectra of $PEA_{0.2}FA_{0.8}SnI_3$ perovskite. The HOMO level of this perovskite corresponds is determined to be -5.06 eV againt the vacuum at 0 eV.

Table S1. Thickness of Sn perovskite with different antisolvents

Solvents	Thickness (nm)
Diisopropyl ether (DIE)	208 ± 5
Diethyl ether (DE)	210 ± 5
Toluene	191 ± 5

Table S2. The FWHM of (001) peak from XRD patterns and crystallite size of Sn perovskite films on glass.

Solvents	FWHM (°)	Crystallite size (nm)
Diisopropyl ether (DIE)	0.11227	70
Diethyl ether (DE)	0.13374	59
Toluene	0.13143	60

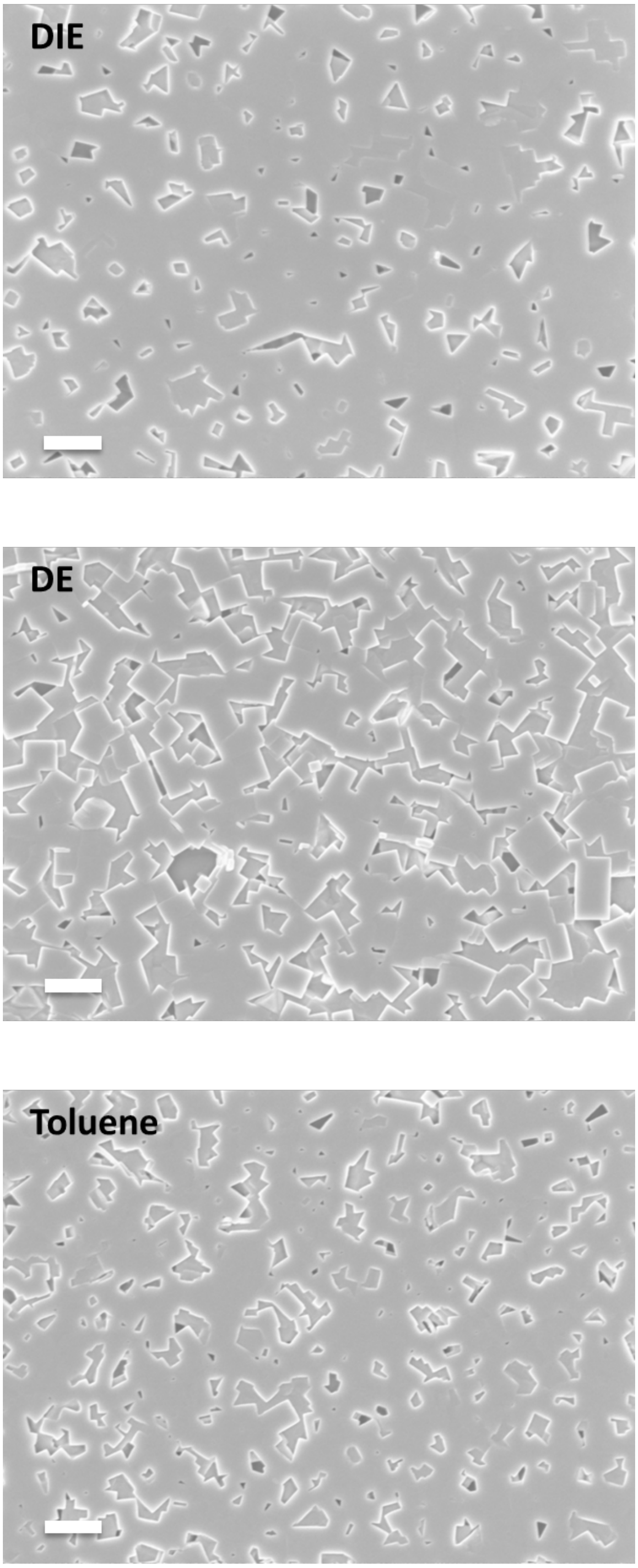

Figure S7. The morphologies of Sn perovskite treated by different antisolvents. The size of the scale bar is 500 nm.

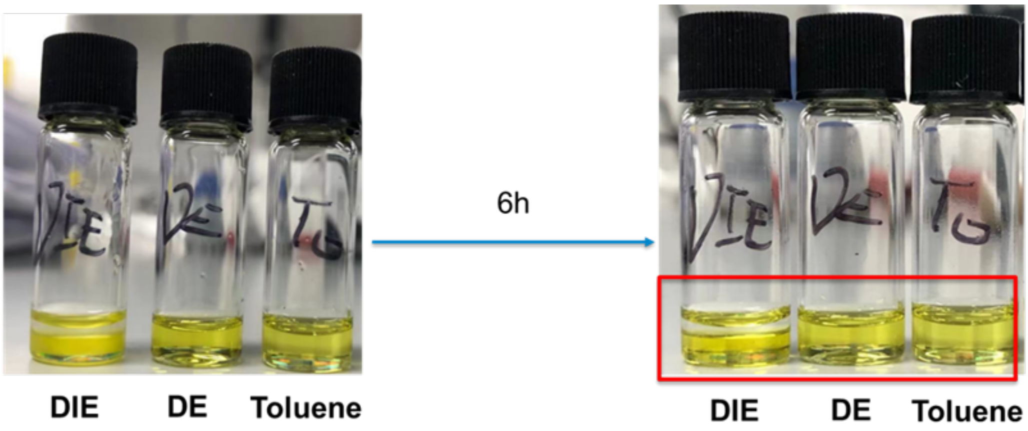

Figure S8. Miscibility study of the perovskite precursor and three different antisolvents. Each vials contains 250 µl pervoskite precursors and 250 µl antisolvents. Photographs taken 0 and 6h after mixing precursors and antisolvents.

Table S3. Physical characteristics of each antisolvents

Solvents	Relative Polarity	Boiling point (°C)
Diisopropyl ether (DIE)	0.110	68.3
Diethyl ether (DE)	0.117	34.6
Toluene	0.099	110.6

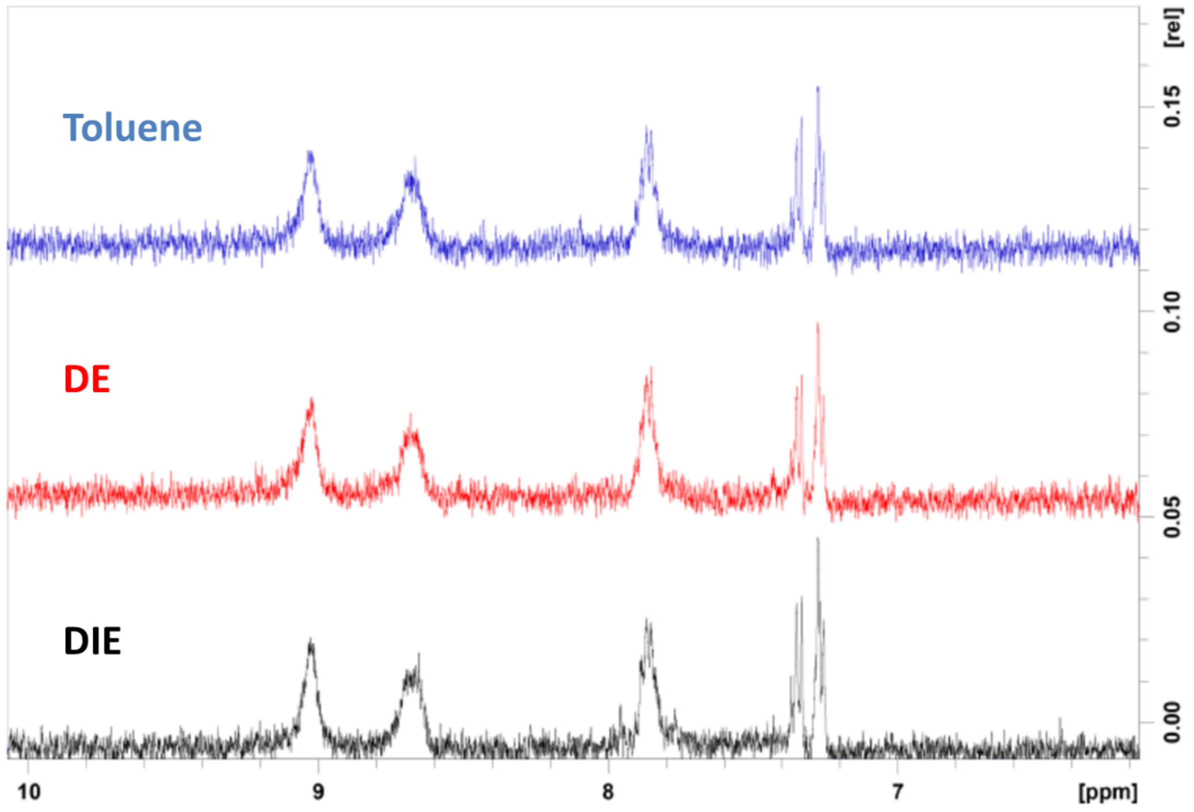

Figure S9. NMR spectra of perovskite films treated by toluene, DE and DIE antisolvents without annealing dissolved in DMSO-d6

In this 1HNMR figure, the peaks located at -9.05 ppm and -8.70 ppm are account for 4 H atoms in FAI and the peaks located around -7.25 ppm and -7.35 ppm are account for 3 atoms in PEAI. Herein, we compared the ratio of integrated peak areas between the 2 peaks of FAI and the 2 peaks of PEAI, shown in Table S3. The results show that DIE treated Sn perovskite has a higher ratio of PEAI inside film, which means more PEA residue after antisolvent treatments.

Table S4. Ratio of the peak areas of FA (at -9.05 ppm and -8.70 ppm) to PEA (-7.35 ppm and -7.25 ppm)

Solvents	Ratio of peak areas (FA: PEA)
Diisopropyl ether (DIE)	1.7564
Diethyl ether (DE)	2.3480
Toluene	2.6177

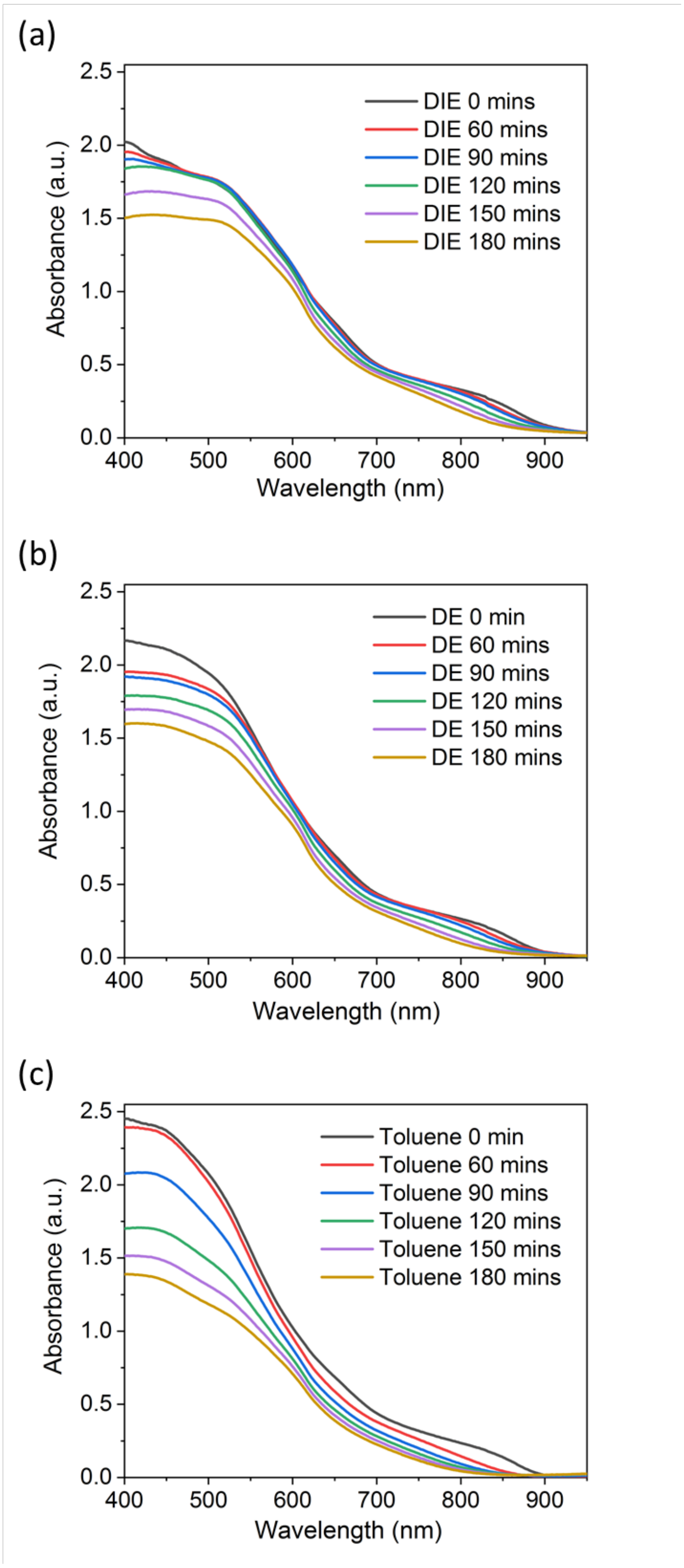

Figure S10. UV-vis spectra of perovskite degradation at ambient atmospheres (a) DIE. (b) DE. (c) Toluene.

Supplementary Note 1: Investigating Changes in Background Hole Density as a Function of O_2 Exposure with Time-Correlated Single Photon Counting Measurements

Recombination in a *p*-doped semiconductor, such as Sn perovskite, can be modelled as follows[1]:

$$-\frac{dn}{dt} = an + bn(p + p_0) \quad (1)$$

Where $\frac{dn}{dt}$ is the recombination rate, a is the rate constant for non-radiative decay of minority (n-type) carriers, b is the rate constant for bimolecular radiative recombination, and *n, p*, and p_0 are the concentrations of photogenerated electrons, photogenerated holes and background holes respectively. Here, the third-order term associated with Auger recombination has been omitted as Auger processes are largely accepted to be irrelevant at low light intensity. Furthermore, in the limit of low excitation fluence ($p << p_0$) (relevant to our time-correlated single photon counting system), this expression simplifies to a pseudo-first order expression:

$$-\frac{dn}{dt} = an + bp_0 n \quad (2)$$

As such, the PL intensity as a function of time, t, after excitation is given by:

$$PL(t) = Ae^{-(a+bp_0)t} = Ae^{-\left(\frac{1}{\tau_m}\right)t} \quad (3)$$

Where τ_m is the measured single exponential lifetime from TCSPC measurements. We can also express the PL intensity from TCSPC in terms of the PL quantum yield (PLQY), *a* and bp_0:

$$PL = C.PLQY = C.\frac{bp_0}{bp_0 + a} \quad (4)$$

Where C is a calibration constant associated with our TCSPC set up. By combining equations (4) and (3), we can obtain the following expression linking the PL intensity and lifetime after a given exposure to p_0:

$$PL_j = C.bp_{0,j}\tau_{m,j} \quad (5)$$

As such, given that C and *b* are constants, we can link the ratio of PL at any given time after oxygen exposure to the initial PL intensity (PL_j/PL_i) with the ratio of background hole densities ($p_{0,j}/p_{0,i}$) and PL decay times ($\tau_{m,j}/\tau_{m,i}$).

$$\frac{PL_j}{PL_i} = \frac{p_{0,j}\tau_{m,j}}{p_{0,i}\tau_{m,i}} \quad (6)$$

Therefore, in this way we can extract relative information about p_0 as a function of time under air exposure. We note that p_0 can be directly estimated if the PLQY and TCSPC could be measured at the same intensity.[2] However, due to the lack overlapping intensity ranges in our TCSPC and PLQY systems, we opted for the method described above.

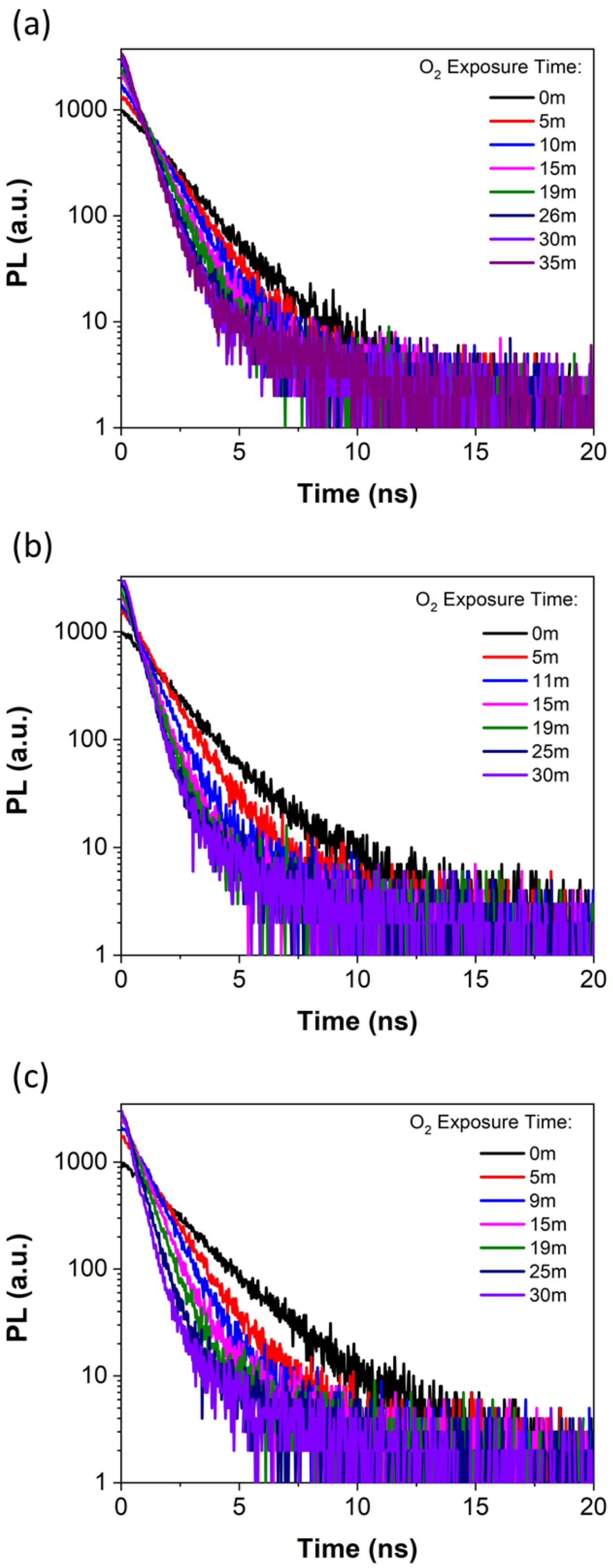

Figure S11. Photoluminescence decays of Sn perovskite treated by (a) DIE, (b) DE and (c) toluene as a function of time under oxygen exposure

References

(1) Milot, R. L.; Klug, M. T.; Davies, C. L.; Wang, Z.; Kraus, H.; Snaith, H. J.; Johnston, M. B.; Herz, L. M. The Effects of Doping Density and Temperature on the Optoelectronic Properties of Formamidinium Tin Triiodide Thin Films. *Adv Mater* **2018**, *30* (44), e1804506. DOI: 10.1002/adma.201804506 From NLM PubMed-not-MEDLINE.
(2) Westbrook, R. J. E.; Taddei, M.; Giridharagopal, R.; Jiang, M.; Gallagher, S. M.; Guye, K. N.; Warga, A. I.; Haque, S. A.; Ginger, D. S. Local Background Hole Density Drives Nonradiative Recombination in Tin Halide Perovskites. *ACS Energy Letters* **2024**, *9* (2), 732-739. DOI: 10.1021/acsenergylett.3c02701.
